# Sevoflurane-Associated Plasma Extracellular Vesicles Promote Aggressive Phenotypes in Cervical Cancer Cells with Concurrent DG Remodeling and EGFR/PKCα/NF-κB Activation

**DOI:** 10.3390/biomedicines14061333

**Published:** 2026-06-12

**Authors:** Bo Jiao, Danning Wang, Jia Wei, Shaodi Guan, Yali Li, Yun Liu, Shaomeng Si, Yueyang Xin, Jie Dong, Siqi Zhou, Pei Lu, Hui Xu

**Affiliations:** 1 Department of Anesthesiology and Pain Medicine, Hubei Key Laboratory of Geriatric Anesthesia and Perioperative Brain Health, and Wuhan Clinical Research Center for Geriatric Anesthesia, Tongji Hospital, Tongji Medical College, Huazhong University of Science and Technology, Wuhan 430030, China; 2024tj0085@hust.edu.cn (B.J.); guanshaodi@yeach.net (S.G.); varawg@163.com (Y.L.); ssmeng0424@163.com (S.S.); king000999@163.com (Y.X.); jayedonne0528@163.com (J.D.); zhousiqi0616@163.com (S.Z.); ludyyi@126.com (P.L.); 2 Department of Anesthesiology, Sir Run Run Shaw Hospital, School of Medicine, Zhejiang University, Hangzhou 310016, China; 3323153@zju.edu.cn; 3 Department of Obstetrics and Gynecology, Tongji Hospital, Tongji Medical College, Huazhong University of Science and Technology, Wuhan 430030, China; jwei@tjh.tjmu.edu.cn; 4 Department of Anesthesiology, Wuhan Fourth Hospital, Wuhan 430030, China; 18771041611@163.com

**Keywords:** sevoflurane, extracellular vesicles, cervical cancer, diacylglycerol, EGFR/PKCα/NF-κB signaling

## Abstract

**Background/Objectives**: Whether anesthetic maintenance influences tumor biology in cervical cancer remains unsettled. We examined whether plasma extracellular vesicles (EVs) collected during sevoflurane or propofol anesthesia differentially affect HeLa cell behavior and explored lipidomic alterations associated with the biologically active EV condition. **Methods**: In a single-center prospective observational cohort, paired plasma samples were collected before anesthesia induction and before wound closure from 53 patients with stage II cervical cancer undergoing radical surgery under sevoflurane (*n* = 28) or propofol (*n* = 25) anesthesia. EV preparations were characterized by transmission electron microscopy, nanoparticle tracking analysis, and immunoblotting for EV markers. Their effects on HeLa cell proliferation, invasion, and wound closure, as well as HUVEC tube formation, were examined in vitro. EV miRNA profiles were analyzed by small-RNA sequencing. Lipidomic profiling by LC-MS and immunoblot analysis of EGFR/PKCα/NF-κB signaling were performed in recipient HeLa cells exposed to sevoflurane-associated EVs. **Results**: EVs collected after sevoflurane anesthesia increased HeLa cell proliferation, invasion, and wound closure and enhanced endothelial branching in HUVEC tube-formation assays, whereas post-propofol EVs showed no comparable phenotype. Small-RNA sequencing identified distinct anesthesia-associated EV miRNA changes, with the sevoflurane-related signature enriched in glycerolipid metabolism, glycerophospholipid metabolism, glycosylphosphatidylinositol-anchor biosynthesis, phosphatidylinositol signaling, and inositol phosphate metabolism. In HeLa cells treated with post-sevoflurane EVs, lipidomic analysis showed clear separation from pre-sevoflurane EV-treated cells and identified increased diacylglycerol (DG) species, including DG (16:1/18:2), DG (16:0/16:1), DG (18:2/18:2), DG (18:2/20:4), and DG (16:0/18:2). These changes were accompanied by higher p-EGFR, PKCα, and p-NF-κB p65 levels. Several DG species correlated positively with proliferation and invasion readouts and inversely with residual wound area. **Conclusions**: Plasma EVs collected after sevoflurane anesthesia were associated with a more aggressive phenotype in recipient cervical cancer cells and with lipid remodeling characterized by DG accumulation and altered EGFR/PKCα/NF-κB signaling. The data support an exploratory mechanistic model linking sevoflurane-associated EV cargo to metabolic reprogramming in cervical cancer cells.

## 1. Introduction

Despite substantial progress in screening, vaccination, and treatment, cervical cancer remains a major cause of cancer-related morbidity and mortality in women worldwide [1]. Surgical resection remains a cornerstone of treatment for resectable disease, yet oncologic outcome is determined not only by tumor stage and pathology but also by perioperative biological events that may influence residual tumor cells and host responses [2,3]. Among perioperative factors, anesthetic maintenance has attracted sustained attention because volatile and intravenous anesthetics may differ in their effects on tumor biology, inflammation, and antitumor immunity [4]. However, clinical evidence comparing these techniques remains inconsistent across tumor types and study designs [5,6]. In gynecologic malignancies, some retrospective data have suggested less favorable long-term outcomes after sevoflurane-based anesthesia than after propofol-based anesthesia, but the biological basis for such observations remains unclear [7].

Extracellular vesicles (EVs) are membrane-enclosed particles released by most cell types and are increasingly recognized as major mediators of intercellular communication [8]. By transferring proteins, nucleic acids, and lipids to recipient cells, EVs can reshape proliferation, invasion, metastasis, angiogenesis, and therapeutic response in cancer [9,10]. EV-associated cargo is particularly relevant in the perioperative setting, where systemic signals generated during anesthesia and surgery may alter the composition and biological activity of circulating vesicles [11]. Whether anesthesia-associated plasma EVs can directly modulate the behavior of cervical cancer cells, however, remains largely unknown.

Lipid metabolism provides a plausible mechanistic link between circulating EVs and tumor progression. EVs can participate in the intercellular transfer of lipids and lipid-related enzymes and can thereby alter membrane composition and signaling in recipient cells [12]. More broadly, metabolic rewiring is a hallmark of malignant growth, and altered lipid metabolism is increasingly recognized as a determinant of tumor cell survival, plasticity, and progression [13,14]. In cervical cancer, lipid dysregulation has clinical and biological relevance. Obesity has been associated with worse outcomes [15], CD36-dependent fatty-acid uptake promotes tumor growth and metastatic behavior [16,17], and modulation of unsaturated fatty-acid signaling can influence treatment response [18]. Together, these observations support the premise that lipid remodeling may serve as an interface between perioperative exposures and cervical cancer biology.

In the present study, we investigated whether plasma EVs collected before and after sevoflurane or propofol anesthesia differentially affect recipient-cell behavior in cervical cancer and whether lipid remodeling is associated with the biologically active EV condition. Using paired perioperative plasma samples from patients with stage II cervical cancer, we combined EV characterization, functional assays in HeLa cells and human umbilical vein endothelial cells (HUVECs), EV miRNA profiling, recipient-cell lipidomics, and signaling analysis. We found that EVs collected after sevoflurane anesthesia were associated with enhanced aggressive phenotypes in recipient cells, lipid metabolic remodeling characterized by diacylglycerol enrichment, and increased EGFR/PKCα/NF-κB signaling. These findings support an exploratory mechanistic framework linking anesthesia-associated circulating EVs to lipid remodeling in cervical cancer.

## 2. Materials and Methods

### 2.1. Design and Participants

The study was conducted in accordance with the Declaration of Helsinki, and the protocol was approved by the Medical Ethics Committee of Tongji Medical College, Huazhong University of Science and Technology (No. S053) on 26 March 2021. Written informed consent was obtained from all participants.

This single-center prospective observational cohort included women aged 35–60 years with pathologically confirmed FIGO stage II cervical cancer, scheduled for radical surgery under general anesthesia. Maintenance anesthesia (sevoflurane or propofol) was selected as part of routine clinical care. A total of 53 eligible patients were enrolled: 28 in the sevoflurane cohort and 25 in the propofol cohort. Baseline demographic, tumor-related, and perioperative characteristics were recorded; the two cohorts were comparable for these variables (Table S1). Patients with neoadjuvant therapy, known distant metastasis, or major organ dysfunction were excluded.

### 2.2. Anesthetic Management and Blood Collection

General anesthesia was induced with etomidate (0.3 mg/kg), sufentanil (0.5 μg/kg), and rocuronium (0.9 mg/kg). After endotracheal intubation, anesthesia was maintained with either sevoflurane inhalation (2%) or continuous intravenous propofol infusion (6–8 mg/kg/h). Remifentanil (0.1–0.15 μg/kg/min) was administered continuously for analgesia, with additional rocuronium as required. Depth of anesthesia and vital signs were monitored continuously. Central venous blood samples were collected into EDTA-containing tubes at two predefined perioperative time points: immediately before anesthesia induction and immediately at the end of surgery, corresponding to approximately 2 to 4 h after anesthesia induction.

### 2.3. Isolation of Plasma EV Preparations

Plasma was obtained by centrifugation of blood at 800× *g* for 10 min at 4 °C. Residual cells and debris were removed by sequential centrifugation at 3000× *g* for 10 min and 10,000× *g* for 30 min at 4 °C. The resulting supernatants were ultracentrifuged at 100,000× *g* for 70 min at 4 °C. The pellets were washed once with phosphate-buffered saline (PBS) and ultracentrifuged again at 100,000× *g* for 70 min at 4 °C. The final pellets were resuspended in PBS.

Differential ultracentrifugation of plasma enriches for extracellular vesicles but may also co-precipitate non-vesicular components, including circulating lipoproteins. These preparations are therefore considered EV-enriched plasma fractions rather than purified EV populations. For readability, they are referred to as EVs after this definition. No additional purification procedures, such as density-gradient ultracentrifugation or size-exclusion chromatography, were applied, and lipoprotein-associated markers such as ApoB were not evaluated. Consequently, co-isolation of lipoprotein particles cannot be formally excluded under the present experimental conditions. All lipidomic and functional analyses were conducted in recipient HeLa cells following exposure to these EV-enriched preparations rather than by direct molecular profiling of the isolated pellets themselves.

EV preparations were classified into four groups according to anesthetic exposure and sampling time: S-Pre-EVs (before sevoflurane), S-Post-EVs (after sevoflurane), P-Pre-EVs (before propofol), and P-Post-EVs (after propofol). Each biological replicate in functional assays, small-RNA sequencing, and lipidomic analyses was derived from an individual patient’s EV preparation.

### 2.4. Transmission Electron Microscopy (TEM)

A drop of EV suspension was placed onto a copper grid and allowed to adsorb for 5 min. Excess liquid was removed with filter paper. The grid was then negatively stained with 2% uranyl acetate for 5 min in the dark, air-dried at room temperature, and examined using a transmission electron microscope (FEI, Hillsboro, OR, USA) to assess EV morphology.

### 2.5. Nanoparticle Tracking Analysis

EV suspensions were diluted in PBS to an appropriate concentration and analyzed using a nanoparticle tracking analysis instrument (ZetaView, Particle Metrix, Meerbusch, Germany) according to the manufacturer’s instructions. Particle size distribution and concentration were calculated on the basis of Brownian motion using the Stokes–Einstein equation.

### 2.6. Western Blotting

For EV characterization and signaling analysis, protein concentrations of EV preparations or cell lysates were measured using a BCA Protein Assay Kit (Boster, Wuhan, China). Equal amounts of protein were separated by SDS-polyacrylamide gel electrophoresis and transferred onto PVDF membranes (Millipore, Billerica, MA, USA). Membranes were blocked with 5% non-fat milk for 1 h at room temperature and then incubated overnight at 4 °C with primary antibodies against Alix (Abcam, Cambridge, UK, ab275377), CD63 (Abcam, Cambridge, UK, ab134045), Tsg101 (Abcam, Cambridge, UK, ab125011), EGFR (ABclonal, Wuhan, China, A11351), phospho-EGFR (Abcam, Cambridge, UK, ab319113), PKCα (ABclonal, Wuhan, China, A11107), NF-κB p65 (Abcam, Cambridge, UK, ab16502), phospho-NF-κB p65 (Cell Signaling Technology, Danvers, MA, USA, 3033), and β-actin (Abcam, Cambridge, UK, ab115777). After incubation with the corresponding secondary antibodies for 1 h at room temperature, signals were visualized by chemiluminescence. Band intensities were quantified by densitometry. Phosphorylated proteins were normalized to their corresponding total proteins, and total protein levels were normalized to β-actin where appropriate.

### 2.7. Cell Culture and EV Treatment

HeLa cells were obtained from the Cell Bank of the Chinese Academy of Sciences (Shanghai, China), and HUVECs were obtained from the American Type Culture Collection (ATCC, Manassas, VA, USA). HeLa cells were cultured in DMEM supplemented with 10% fetal bovine serum and 1% penicillin/streptomycin at 37 °C in a humidified atmosphere containing 5% CO_2_. HUVECs were cultured in Endothelial Cell Growth Medium-2 (EGM-2; Lonza, Walkersville, MD, USA) supplemented with the EGM-2 SingleQuots™ kit according to the manufacturer’s instructions. For functional experiments, cells were treated with PBS or EVs at a final concentration of 250 μg/mL.

### 2.8. EV Uptake Assay

EVs were labeled with PKH26 to examine cellular uptake. Briefly, 100 μL EV suspension (100 μg/mL) was resuspended in 1 mL PBS and incubated with 4 μL PKH26 dye (Sigma-Aldrich, St. Louis, MO, USA) for 20 min at 37 °C. Labeled EVs were washed twice by ultracentrifugation at 100,000× *g* for 70 min at 4 °C to remove excess dye and were then resuspended in PBS. HeLa cells were incubated in serum-free medium until attachment and then exposed to PKH26-labeled EVs for 12 h. After incubation, cells were washed with PBS, fixed with 4% paraformaldehyde, counterstained with Hoechst 33342, and examined by confocal fluorescence microscopy (Leica Microsystems, Wetzlar, Germany).

### 2.9. CCK-8 Proliferation Assay

HeLa cells were seeded into 96-well plates in technical triplicate at a density of 5 × 10^3^ cells per well. After attachment, cells were treated with PBS or EVs for 24, 48, or 72 h. The culture medium was then replaced with DMEM containing 10% Cell Counting Kit-8 reagent (MCE, Monmouth Junction, NJ, USA), and cells were incubated for 1 h at 37 °C. Absorbance at 450 nm was measured using a microplate reader (BioTek, Winooski, VT, USA).

### 2.10. Wound-Healing Assay

HeLa cells were seeded into 6-well plates in technical triplicate and cultured to near confluence. A straight scratch was created using a sterile pipette tip, and detached cells were removed by washing with PBS. Cells were then cultured in low-serum medium containing PBS or EVs. Images were obtained at 0, 12, and 24 h using an inverted microscope (Olympus, Tokyo, Japan). Residual wound area was quantified using ImageJ (version 1.52) and expressed relative to the wound area at 0 h.

### 2.11. Transwell Invasion Assay

HeLa cells were seeded into Matrigel-coated Transwell inserts (Corning, Corning, NY, USA) at 2 × 10^4^ cells per well. After attachment, cells in the upper chamber were treated with PBS or EVs (250 μg/mL). The lower chamber was filled with DMEM containing 10% fetal bovine serum as a chemoattractant. After 24 h of incubation at 37 °C, cells remaining on the upper surface of the membrane were removed. Cells that had traversed to the lower surface were fixed with 4% paraformaldehyde for 20 min, stained with 0.1% crystal violet for 10 min, washed with PBS, air-dried, and visualized under a light microscope (Olympus, Tokyo, Japan).

### 2.12. Tube-Formation Assay

Matrigel matrix (BD, Franklin Lakes, NJ, USA) was added to 96-well plates and allowed to polymerize at 37 °C for 45 min. HUVECs were then seeded onto the gel at 2.5 × 10^4^ cells per well in technical triplicate and treated with PBS or EVs. After 6 h of incubation at 37 °C in 5% CO_2_, tube-like structures were imaged under a light microscope (Olympus, Tokyo, Japan). Branch points were quantified using ImageJ.

### 2.13. Small-RNA Sequencing and Bioinformatic Analysis

Total RNA was extracted from EV preparations using TRIzol reagent (Invitrogen, Carlsbad, CA, USA) according to the manufacturer’s instructions. RNA concentration was measured using an ND-2000 spectrophotometer (NanoDrop Technologies, Wilmington, DE, USA), and RNA quality was assessed using an Agilent 2100 Bioanalyzer (Agilent Technologies, Palo Alto, CA, USA). Small-RNA libraries were prepared from 3 μg total RNA using the TruSeq Small RNA Sample Prep Kit (Illumina, San Diego, CA, USA). After library amplification (11–12 PCR cycles), sequencing was performed on the Illumina HiSeq X Ten/NovaSeq 6000 platform (Illumina, San Diego, CA, USA).

After adaptor trimming and quality control, reads were mapped to miRBase version 21.0 to generate miRNA expression profiles. miRNA abundance was expressed as transcripts per million. Differential expression analysis was performed using DESeq2 (version 1.42.0), and miRNAs with |log2 fold change| > 1.2 and a false discovery rate (FDR) < 0.05 were considered significantly differentially expressed. Predicted target genes of differentially expressed miRNAs were subjected to Kyoto Encyclopedia of Genes and Genomes (KEGG) enrichment analysis, and the top enriched pathways were visualized as bubble plots.

### 2.14. RT-qPCR Validation of Selected EV miRNAs

To technically validate the small-RNA sequencing results, candidate miRNAs from the sevoflurane comparison were prioritized for RT-qPCR on the basis of concordant directional change across sequenced biological replicates and relatively high abundance in the sequencing dataset, which favored robust detectability. Total RNA was reverse-transcribed using a miRNA-specific reverse transcription system according to the manufacturer’s instructions. Quantitative PCR was performed on a real-time PCR platform (Bio-Rad, Hercules, CA, USA) using SYBR Green Master Mix (Thermo Fisher Scientific, Waltham, MA, USA). Relative miRNA expression was calculated using the 2^−ΔΔCt^ method after normalization to U6 snRNA. All reactions were run in technical triplicate. Primer sequences are listed in Table S2.

### 2.15. Lipidomic Analysis by LC-MS

HeLa cells treated with S-Pre-EVs or S-Post-EVs were washed with PBS, scraped into 1.5-mL tubes, and pelleted by centrifugation. Cell numbers were normalized before extraction. Samples were homogenized with beads, extracted with an organic solvent mixture, sonicated at −10 °C, and centrifuged. The resulting supernatants were transferred to LC-MS autosampler vials (Thermo Fisher Scientific, Waltham, MA, USA) for analysis.

Chromatographic separation was performed on an HSS T3 column maintained at 40 °C at a flow rate of 0.40 mL/min using an acetonitrile/water-based mobile phase containing formic acid. Mass spectrometric data were acquired in both positive and negative ion modes over an *m*/*z* range of 70–1050 with high-resolution MS1 and MS2 acquisition. Lipid feature annotation was performed with reference to the Human Metabolome Database (HMDB, version 4.0) and KEGG.

### 2.16. Statistical Analysis

Statistical analyses were performed using GraphPad Prism 9.5, SPSS 27.0, and R 4.2.0. Continuous clinical variables were tested for normality using the Shapiro–Wilk test and are presented as mean ± standard deviation or median (interquartile range), as appropriate. Categorical variables are presented as counts and percentages. Baseline clinical characteristics between the sevoflurane and propofol cohorts were compared using Student’s *t* test or Mann–Whitney U test for continuous variables and the chi-square test or Fisher’s exact test for categorical variables.

For cell-based and molecular experiments, each biological replicate represented an independent patient-derived EV preparation. Technical triplicates were averaged before statistical testing. Data are presented as mean ± standard error of the mean unless otherwise specified. Time-course experiments, including CCK-8 and wound-healing assays, were analyzed using two-way ANOVA with treatment group and time as factors. Post hoc comparisons were performed using Bonferroni correction to assess simple effects within each time point. Single-time-point assays involving more than two groups were analyzed by one-way ANOVA followed by Bonferroni post hoc test.

DFS and OS were estimated using the Kaplan–Meier method and compared with the log-rank test; these analyses were exploratory. For small-RNA sequencing, differential expression was analyzed with DESeq2, and miRNAs with |log2 fold change| > 1.2 and FDR < 0.05 were considered differentially expressed. Lipidomic data were subjected to hierarchical clustering and orthogonal partial least squares-discriminant analysis (OPLS-DA), and model robustness was assessed by permutation testing. Differential lipids were defined by a variable importance in projection value >1 and *p* < 0.05. Associations between DG abundance and phenotypic readouts were examined using Spearman rank correlation. All tests were two-sided, and *p* < 0.05 was considered statistically significant.

Unless otherwise specified, three independent EV preparations per group were used for functional assays and sequencing, and six per group for lipidomic analysis.

## 3. Results

### 3.1. Clinical Characteristics of the Study Cohort

53 patients met the eligibility criteria and were included in the analysis, comprising 28 patients in the sevoflurane cohort and 25 patients in the propofol cohort. The 2 cohorts were comparable with respect to age, body mass index, stage II subtype distribution, preoperative squamous cell carcinoma antigen level, duration of anesthesia, perioperative opioid exposure, fluid administration, and minimum intraoperative body temperature (Table S1). Paired plasma samples were collected before anesthesia induction and before wound closure in all participants for downstream EV isolation.

Exploratory follow-up over 1500 days identified 4 recurrences and 4 deaths in the entire cohort. Kaplan–Meier analysis showed no significant difference in disease-free survival or overall survival between the sevoflurane and propofol groups (Figure S1A,B).

### 3.2. Characterization and Cellular Uptake of Plasma EVs

**Figure 1:**
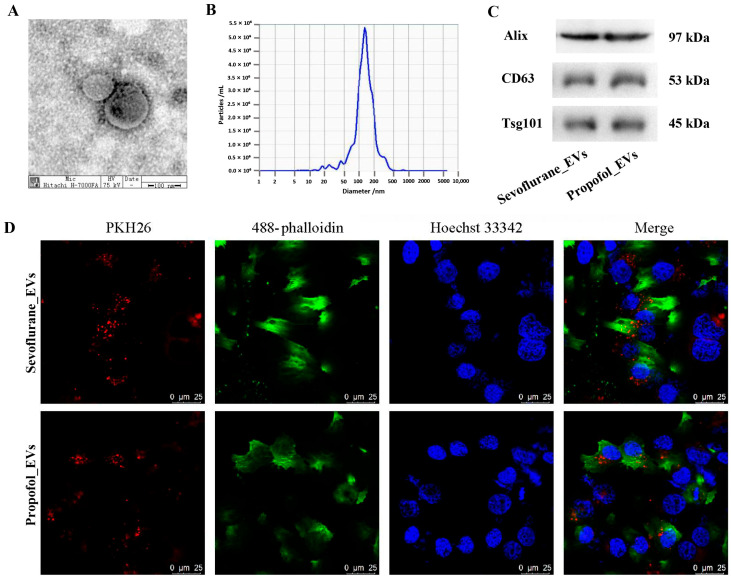
Characterization and tracing of plasma-derived extracellular vesicles (EVs) from cervical cancer patients. (**A**) TEM images showing EVs with bilayer membrane structures (scale bar: 100 nm). (**B**) Nanoparticle tracking analysis (NTA) of EV size distribution (mean diameter: 125.8 nm). (**C**) Western blot confirming EV markers (Alix, CD63, Tsg101) in isolated plasma EVs. (**D**) Tracing of PKH26-labeled EVs (red) in HeLa cells. Cells were counterstained with Hoechst 33342 (nuclei, blue) and 488-phalloidin (cytoskeleton, green) to visualize cellular structure (scale bar: 25 μm).

Plasma EVs isolated by differential ultracentrifugation showed vesicles with the expected membrane-bound morphology on TEM (Figure 1A). Nanoparticle tracking analysis demonstrated a particle-size distribution centered in the small-vesicle range, with a mean diameter of 125.8 nm (Figure 1B). Immunoblotting confirmed the presence of the EV-associated markers Alix, CD63, and Tsg101 in the isolated preparations (Figure 1C).

To examine whether these plasma EVs interacted with recipient tumor cells, PKH26-labeled EVs were incubated with HeLa cells. Punctate intracellular red fluorescence was observed after incubation, consistent with cellular uptake under the present experimental conditions (Figure 1D).

### 3.3. Post-Sevoflurane EVs Enhance Aggressive Phenotypes in HeLa Cells and Proangiogenic Responses in HUVECs

**Figure 2:**
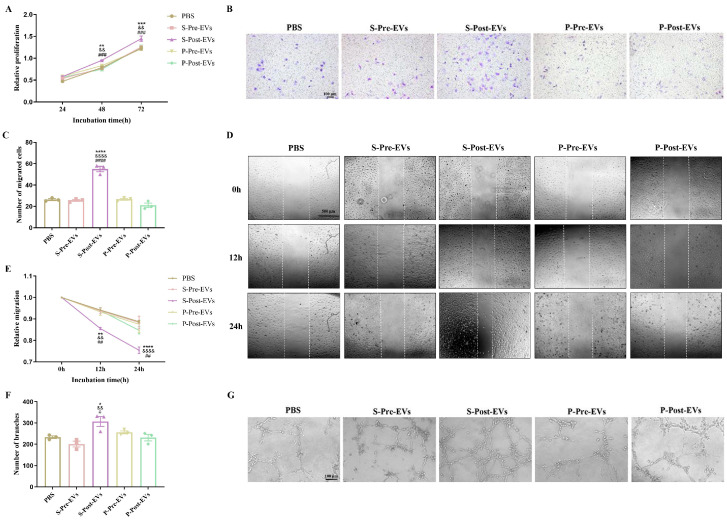
Functional effects of post-sevoflurane EVs on HeLa cells and HUVECs. (**A**) HeLa cell proliferation assessed by CCK-8 assay at 24, 48, and 72 h. (**B**,**C**) Transwell invasion assay with representative images and quantification of invaded HeLa cells (scale bar: 100 μm). (**D**,**E**) Wound-healing assay with representative images and quantification of residual wound area at 12 and 24 h (scale bar: 500 μm). (**F**,**G**) HUVEC tube-formation assay with representative images and quantification of branch points (scale bar: 100 μm). Data are presented as mean ± SEM. Each biological replicate represented an independent patient-derived EV preparation (*n* = 3 per group); technical triplicate wells were averaged before analysis. * *p* < 0.05, ** *p* < 0.01, *** *p* < 0.001, **** *p* < 0.0001 vs. PBS group; && *p* < 0.01, &&&& *p* < 0.0001 vs. S-Pre-EVs group, # *p* < 0.05, ## *p* < 0.01, ### *p* < 0.001, #### *p* < 0.0001 vs. P-Post-EVs group, *n* =  3 in each group, two-way ANOVA, followed by Bonferroni tests.

The biological effects of perioperative plasma EV preparations were evaluated in HeLa cells and HUVECs using five treatment conditions: PBS, S-Pre-EVs, S-Post-EVs, P-Pre-EVs, and P-Post-EVs. In the CCK-8 assay, no clear between-group difference was observed at 24 h; however, by 48 h and 72 h, HeLa cells exposed to S-Post-EVs showed significantly higher proliferative activity than cells treated with PBS, S-Pre-EVs, or P-Post-EVs (Figure 2A).

A similar pattern was observed in the invasion assay. S-Post-EVs produced the highest number of invaded HeLa cells among all treatment groups, whereas P-Post-EVs did not differ materially from P-Pre-EVs or PBS (Figure 2B,C). In the wound-healing assay, S-Post-EV-treated cells exhibited faster wound closure, reflected by a smaller residual wound area at 12 h and 24 h than that observed in the comparator groups (Figure 2D,E). In HUVEC tube-formation assays, S-Post-EVs increased the number of branch points and promoted the formation of more complex capillary-like structures, again without a comparable effect in the post-propofol group (Figure 2F,G).

Taken together, these data showed that the phenotype-positive signal was confined to EVs collected after sevoflurane anesthesia. Because post-propofol EVs did not induce measurable changes in the functional assays used here, subsequent mechanistic analyses were focused on the sevoflurane arm.

### 3.4. Sevoflurane-Associated EV miRNA Alterations Are Enriched in Lipid-Metabolism Pathways

**Figure 3:**
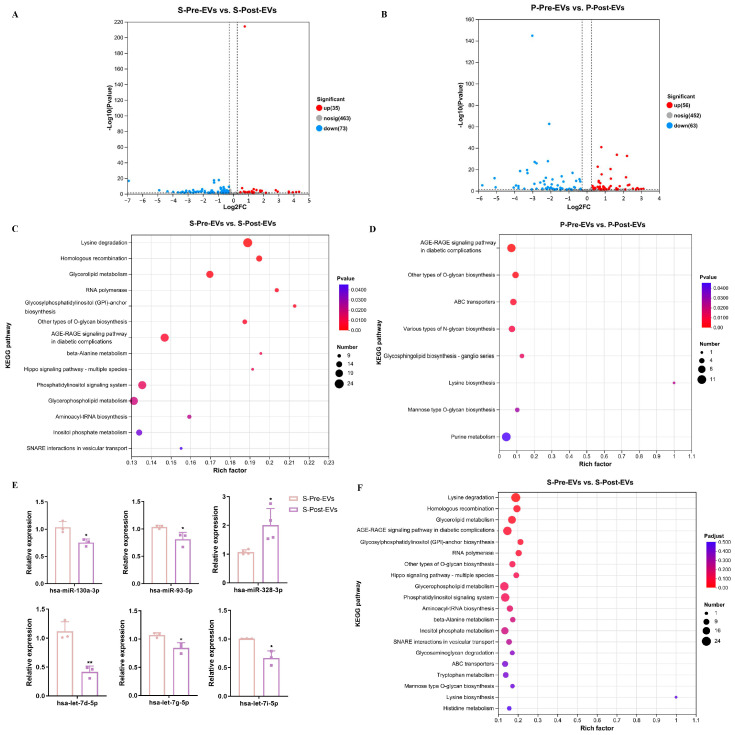
Sevoflurane-associated plasma EV miRNA profiles are enriched in lipid-metabolism pathways. (**A**,**B**) Volcano plots showing differentially expressed miRNAs in plasma EVs from the sevoflurane group (**A**) and the propofol group (**B**), comparing post-anesthesia samples with their respective pre-anesthesia baselines. Red dots indicate significantly upregulated miRNAs, whereas blue dots indicate significantly downregulated miRNAs (|log2 fold change| > 1.2, FDR < 0.05). (**C**,**D**) KEGG pathway enrichment analysis of predicted target genes of differentially expressed miRNAs in the sevoflurane group (**C**) and the propofol group (**D**). (**E**) RT-qPCR validation of six representative miRNAs from the sevoflurane comparison, selected on the basis of concordant directional change across sequenced samples and relatively high abundance in the small-RNA sequencing dataset, in independent EV preparations from S-Pre-EVs and S-Post-EVs (*n* = 3 independent patient-derived EV preparations per group). (**F**) KEGG enrichment plot for the sevoflurane group ranked by adjusted *p* value. Data in (**E**) are presented as mean ± SD. * *p* < 0.05, ** *p* < 0.01 versus S-Pre-EVs.

Small-RNA sequencing identified 571 EV-associated miRNAs across the study samples. Using the predefined threshold of |log2 fold change| > 1.2 with FDR < 0.05, comparison of post- versus pre-anesthesia EVs in the sevoflurane cohort identified 108 differentially expressed miRNAs, including 35 upregulated and 73 downregulated transcripts (Figure 3A). In the propofol cohort, 119 miRNAs were differentially expressed, including 56 upregulated and 63 downregulated transcripts (Figure 3B).

KEGG analysis of predicted target genes showed that the sevoflurane-associated miRNA signature was prominently enriched in pathways related to glycerolipid metabolism, glycerophospholipid metabolism, glycosylphosphatidylinositol-anchor biosynthesis, the phosphatidylinositol signaling system, and inositol phosphate metabolism (Figure 3C). By contrast, the propofol-associated enrichment profile was broader and did not display the same concentration in lipid-centered pathways that characterized the sevoflurane comparison (Figure 3D).

Because the pro-aggressive phenotype in the functional assays was confined to S-Post-EV-enriched preparations, RT-qPCR validation was focused on representative sevoflurane-associated EV miRNAs selected for the mechanistic follow-up. To validate the sequencing findings, we prioritized candidate miRNAs from the sevoflurane comparison on the basis of concordant directional change across sequenced samples and relatively high abundance in the small-RNA sequencing dataset. RT-qPCR confirmed significant downregulation of hsa-let-7d-5p, hsa-let-7g-5p, hsa-let-7i-5p, hsa-miR-130a-3p, and hsa-miR-93-5p, together with significant upregulation of hsa-miR-328-3p, in S-Post-EVs relative to S-Pre-EVs (Figure 3E). These results were directionally consistent with the sequencing data and supported the robustness of the sevoflurane-associated EV-miRNA profile. KEGG enrichment analysis further confirmed glycerolipid metabolism as a significant feature of the sevoflurane group (Figure 3F). Given the pivotal role of lipid signaling in cancer progression, these data collectively nominated lipid remodeling as the most biologically relevant downstream process for further interrogation.

### 3.5. Post-Sevoflurane EVs Remodel the Lipidomic Landscape of HeLa Cells

**Figure 4:**
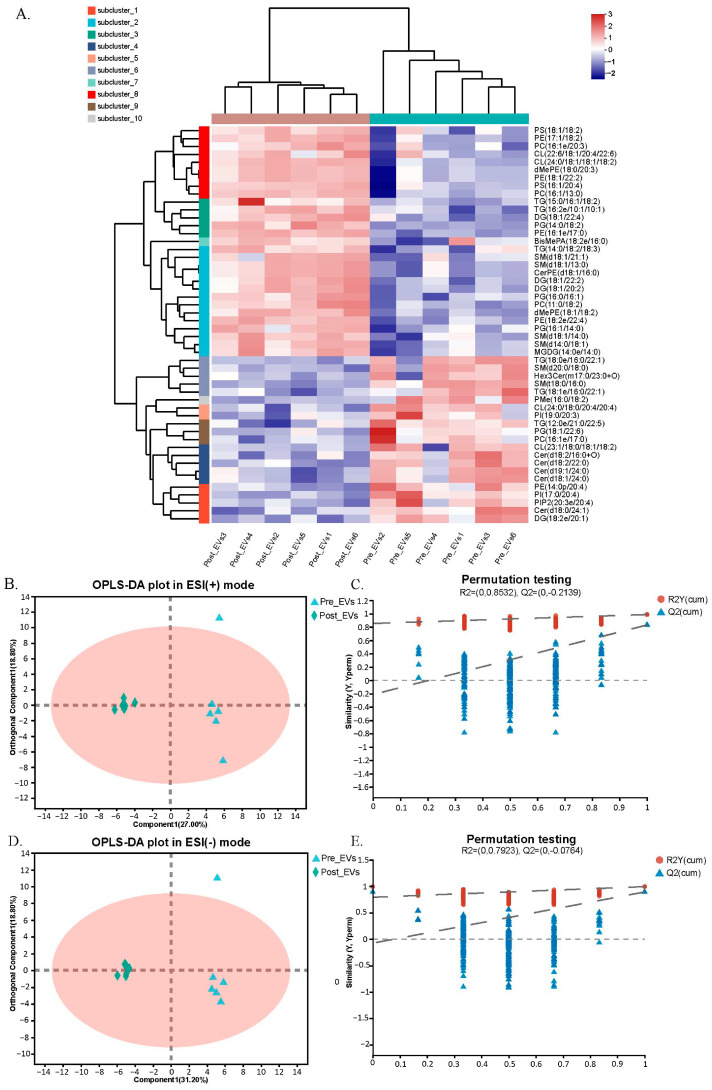
Lipidomic profiling of HeLa cells treated with sevoflurane-primed EVs. (**A**) Hierarchical clustering of lipid profiles (S-Post-EVs vs. S-Pre-EVs). (**B**–**E**) OPLS-DA score plots under (**B**) ESI(+) and (**D**) ESI(−) modes, with permutation tests (**C**,**E**) validating model robustness (Q2 intercept < 0).

On the basis of the functional and miRNA data, lipidomic profiling was performed in HeLa cells exposed to S-Pre-EVs or S-Post-EVs. Unsupervised hierarchical clustering separated the two conditions into distinct groups, indicating a broad shift in the lipid composition of recipient cells after exposure to post-sevoflurane EVs (Figure 4A).

Supervised OPLS-DA further demonstrated clear discrimination between S-Pre-EV-treated and S-Post-EV-treated cells in both ESI(+) and ESI(−) modes, with no meaningful overlap between the two groups (Figure 4B,D). Permutation testing yielded negative Q2 intercepts in both ionization modes, supporting model stability and arguing against overfitting (Figure 4C,E). These results indicated that post-sevoflurane EV exposure was associated with reproducible lipidomic remodeling in recipient HeLa cells.

### 3.6. Differential Lipids in Hela Cells Converge on DG Enrichment and Altered EGFR/PKCα/NF-κB Signaling

**Figure 5:**
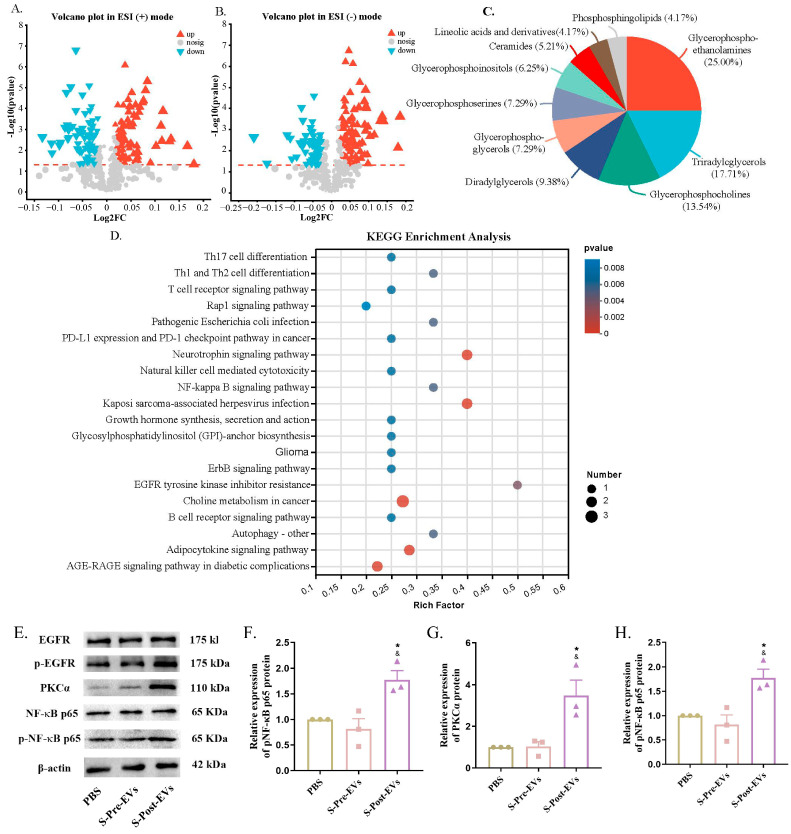
Sevoflurane-EV-driven lipid remodeling activates oncogenic signaling. (**A**,**B**) Volcano plots of differentially expressed lipids in (**A**) ESI(+) and (**B**) ESI(−) modes (*p* < 0.05, VIP > 1). (**C**) Composition of lipid subclasses (Values are rounded to two decimal places; the sum may not be 100% due to rounding). (**D**) KEGG pathways enriched by differential lipids. (**E**–**H**) Western blot quantification of p-EGFR, PKCα, and p-NF-κB p65 expression (* *p* < 0.05 compared with PBS group; & *p* < 0.05 compared with S-Pre-EVs group, *n* = 3 in each group, one-way ANOVA followed by Bonferroni post hoc test).

A total of 1155 lipid species were identified in recipient HeLa cells following exposure to EV-enriched plasma preparations, including 643 species in ESI(+) mode and 512 species in ESI(−) mode. Using VIP > 1 and *p* < 0.05 as the selection criteria, 173 differential lipids were detected in ESI(+) mode, comprising 101 increased and 72 decreased species in the S-Post-EV condition relative to the S-Pre-EV condition (Figure 5A). In ESI(−) mode, 176 differential lipids were identified, including 99 increased and 77 decreased species (Figure 5B).

The altered lipids were distributed across 10 subclasses, of which glycerophosphoethanolamines, triacylglycerols, glycerophosphocholines, and diacylglycerols accounted for the largest fractions (Figure 5C). KEGG pathway analysis linked the differential lipid set to several cancer-relevant pathways, including EGFR tyrosine kinase inhibitor resistance, choline metabolism in cancer, the NF-κB signaling pathway, and the ErbB signaling pathway (Figure 5D).

To determine whether these lipid changes were accompanied by changes in signaling output, immunoblotting was performed in HeLa cells treated with PBS, S-Pre-EVs, or S-Post-EVs. Exposure to S-Post-EVs increased p-EGFR, PKCα, and p-NF-κB p65 relative to both PBS and S-Pre-EVs (Figure 5E–H). These findings connected the lipidomic shift induced by post-sevoflurane EVs with a signaling profile consistent with enhanced malignant behavior.

### 3.7. Increased DG Species Correlate with Proliferation, Invasion, and Wound-Closure Readouts

**Figure 6:**
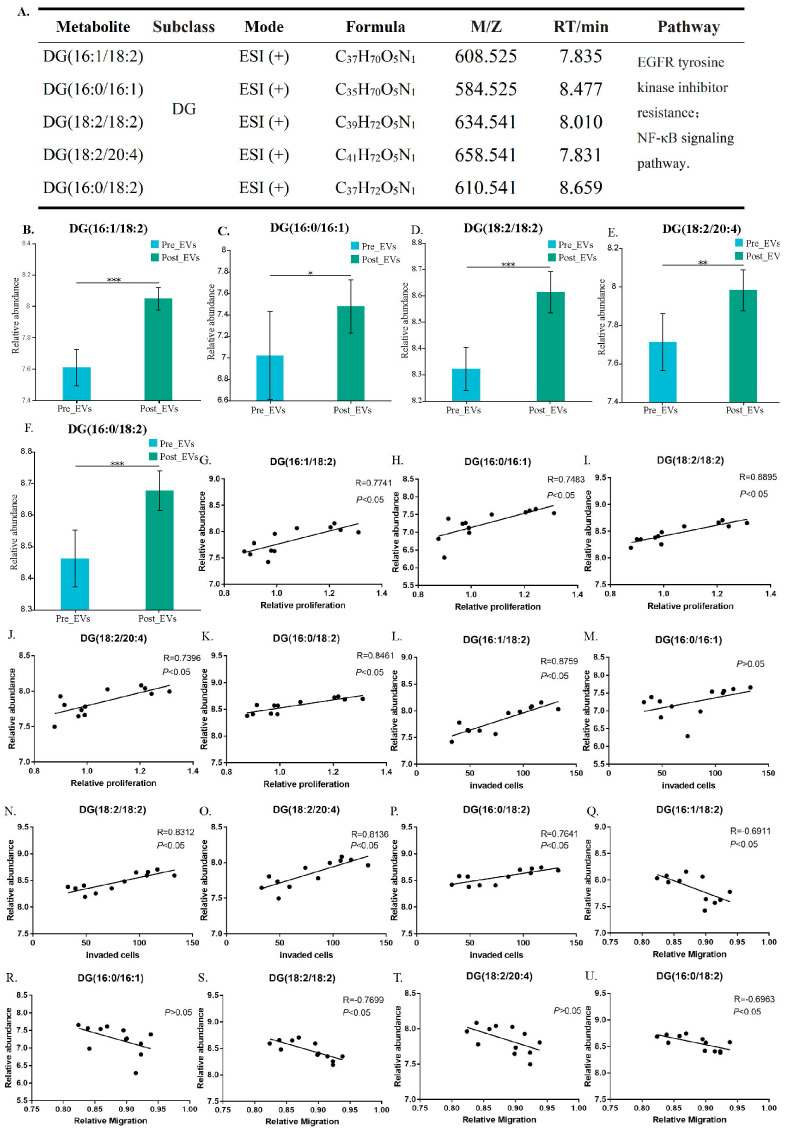
Correlation of diacylglycerol (DG) species with HeLa cell malignancy. (**A**) Five differentially abundant DG species. (**B**–**F**) Relative abundance of DG (16:1/18:2), DG (16:0/16:1), DG (18:2/18:2), DG (18:2/20:4), and DG (16:0/18:2) (* *p* < 0.05, ** *p* < 0.01, *** *p* < 0.001 compared with S-Pre-EVs group). (**G**–**U**) Correlation plots between DG levels and (**G**–**K**) proliferation, (**L**–**P**) invasion, and (**Q**–**U**) migration.

Because DGs were among the lipid subclasses altered in recipient HeLa cells following exposure to EV-enriched plasma preparations and were annotated to pathways linked to EGFR tyrosine kinase inhibitor resistance and NF-κB signaling, this subclass was examined in greater detail (Figure 6A). Five DG species were significantly increased in HeLa cells exposed to S-Post-EVs compared with S-Pre-EVs: DG (16:1/18:2), DG (16:0/16:1), DG (18:2/18:2), DG (18:2/20:4), and DG (16:0/18:2) (Figure 6B–F).

Correlation analysis showed that all five DG species were positively associated with proliferative activity in HeLa cells (Figure 6G–K). Among these, DG (18:2/18:2) showed the strongest association with proliferation. Four DG species—DG (16:1/18:2), DG (18:2/18:2), DG (18:2/20:4), and DG (16:0/18:2)—were positively correlated with invaded cell number, whereas DG (16:0/16:1) was not significantly associated with invasion (Figure 6L–P). Residual wound area was inversely correlated with DG (16:1/18:2), DG (18:2/18:2), and DG (16:0/18:2), indicating faster wound closure at higher DG abundance, while no significant association was observed for DG (16:0/16:1) or DG (18:2/20:4) (Figure 6Q–U). These data identified DG enrichment as the lipid feature most consistently linked to the aggressive cellular phenotype induced by post-sevoflurane EVs.

## 4. Discussion

This study identified a consistent EV-associated biological signal linked to sevoflurane anesthesia in cervical cancer. Among the perioperative EV conditions tested, only EVs collected after sevoflurane anesthesia reproducibly enhanced HeLa cell proliferation, invasion, and wound closure and increased endothelial branching in HUVECs. That phenotype was accompanied by a distinct EV miRNA profile enriched in lipid-related pathways, a broad shift in the lipid composition of recipient HeLa cells, accumulation of several DG species, and increased p-EGFR, PKCα, and p-NF-κB p65. Considered together, these findings define a coherent exploratory model in which sevoflurane-associated circulating EVs are linked to lipid-metabolic remodeling and a more aggressive cell phenotype.

The question of whether anesthetic technique influences long-term cancer outcomes remains unresolved, particularly when retrospective studies, tumor-specific cohorts, and broader comparative analyses are considered together [2,3,5,6,7]. In the present cohort, exploratory follow-up did not show a difference in DFS or OS between the sevoflurane and propofol groups. This finding should not be interpreted as evidence of equivalence between anesthetic techniques, because the study was not powered for oncologic outcome comparisons and only a small number of events occurred across the entire cohort. Accordingly, the present dataset does not permit inference regarding long-term prognosis. The principal value of the clinical cohort lies in the paired perioperative biospecimens, which enabled mechanistic interrogation of anesthesia-associated EV biology under clinically relevant conditions.

EVs are plausible mediators of perioperative biological effects because they transfer proteins, nucleic acids, and lipids between cells and can durably alter recipient-cell behavior [8,9,10,12,19]. The present data add cervical cancer-specific evidence to that concept. Under the conditions tested here, the phenotype-positive signal was confined to the post-sevoflurane EV condition. Post-propofol EVs did not reproduce the same effects in HeLa proliferation, invasion, wound-closure, or HUVEC branching assays. This contrast is important for two reasons. First, it provided the rationale for focusing downstream mechanistic analyses on the sevoflurane arm. Second, it suggests that the perioperative signal detected in this study was not captured equally by the two anesthetic-associated EV states examined here. The increase in HUVEC branching also indicates that the effect was not restricted to tumor cells alone, but extended to a surrogate endothelial readout relevant to angiogenesis.

A related question is why post-propofol EVs did not elicit comparable phenotypic changes in the present assays. Several non-mutually-exclusive possibilities exist. First, sevoflurane and propofol have distinct effects on cellular stress responses, inflammation, and the tumor microenvironment [20,21]. Given that these processes can regulate EV biogenesis and cargo sorting, it is plausible that the two anesthetics differentially influence the composition of circulating EVs. Supporting this concept, Buschmann et al. reported that sevoflurane and propofol differentially alter miRNA cargo in circulating EVs during colorectal cancer resection [22]. In addition, Gluth et al. demonstrated that EV concentration and EV-associated miRNA profiles differ between propofol- and sevoflurane-based anesthesia in bladder cancer patients undergoing radical cystectomy [23]. Similarly, a recent study in hepatocellular carcinoma reported that sevoflurane-associated plasma exosomal miRNA alterations were linked to immune-related biological processes and tumor progression through bioinformatic pathway analyses [24]. Collectively, these studies indicate that anesthetic techniques can influence both the composition and biological relevance of circulating EVs. Consistent with these findings, our small-RNA sequencing data revealed that the sevoflurane-associated EV-miRNA signature was prominently enriched in lipid-metabolism-related pathways, whereas the propofol-associated signature was not. This difference in pathway enrichment may help explain why post-propofol EVs did not produce the same enhancing effects on HeLa cell proliferation, invasion, and wound closure as observed with post-sevoflurane EVs. Importantly, while previous studies primarily inferred potential biological implications from EV-miRNA profiling, the present study integrates EV-miRNA sequencing with functional assays, recipient-cell lipidomics, and signaling analyses, thereby providing complementary evidence that anesthesia-associated EV alterations may be accompanied by measurable phenotypic consequences in recipient cells. Second, the absence of detectable effects in proliferation, invasion, wound closure, or tube formation assays does not exclude the possibility that post-propofol EVs may influence other aspects of tumor biology, such as immune modulation or therapy resistance, which were not evaluated in this study. Therefore, while the present data support focusing mechanistic investigations on the sevoflurane-associated EV effects, the propofol findings should be interpreted as context-dependent and limited to the endpoints assessed here rather than evidence of biological inactivity. Future in vivo studies will be valuable for determining the biological significance of these EV-associated differences in more physiologically relevant settings.

Further analysis of the sevoflurane-associated EV-miRNA signature provided insight into the potential downstream mechanisms. Both anesthetic regimens altered EV miRNA profiles, but the sevoflurane-associated signature was preferentially linked to glycerolipid metabolism, glycerophospholipid metabolism, GPI-anchor biosynthesis, the phosphatidylinositol signaling system, and inositol phosphate metabolism. The concordance between small-RNA sequencing and RT-qPCR for selected miRNAs further supports the robustness of the sevoflurane-associated EV-miRNA signal. RT-qPCR was used as a targeted technical validation of a representative subset of sevoflurane-associated EV miRNAs, rather than as functional evidence for individual miRNAs. Candidate miRNAs were prioritized according to concordant directional change across sequenced samples and relatively high abundance in the sequencing dataset, which favored reliable RT-qPCR detection. The validated subset reproduced the sequencing-defined direction of change and therefore strengthened confidence in the sevoflurane-associated EV-miRNA signal. Because post-propofol EVs did not produce measurable phenotypic effects and downstream mechanistic analyses were focused on the phenotype-positive sevoflurane arm, individual propofol-associated miRNAs were not independently validated in the present study. This enrichment pattern was biologically meaningful because it was mirrored by the lipidomic data obtained in recipient HeLa cells. Cells exposed to post-sevoflurane EVs separated clearly from the pre-sevoflurane condition in both unsupervised clustering and supervised OPLS-DA, indicating global lipidomic remodeling rather than isolated changes in a few metabolites. The continuity from EV-miRNA pathway enrichment to recipient-cell lipid remodeling strengthens the internal logic of the dataset. It is also compatible with current evidence that dysregulated lipid handling contributes to cervical cancer progression, including associations with obesity, altered circulating lipid profiles, fatty-acid uptake, and lymphatic metastatic behavior [15,16,17,18,25,26,27].

Within the altered lipidome, DG species represented the most coherent and biologically interpretable signal. Five DG molecules were increased in HeLa cells after exposure to post-sevoflurane EVs, and the abundance of these species tracked with the major phenotypic readouts. All five correlated positively with proliferation; four correlated positively with invaded cell number; and three were inversely correlated with residual wound area, consistent with faster wound closure at higher DG abundance. These associations do not by themselves establish causality, but they are not random. DG is a signaling lipid with a well-established role in recruiting and activating conventional PKC isoforms at cellular membranes [28]. In cervical neoplasia, altered glycerolipid composition has also been reported at the plasma level [27], making DG enrichment a biologically credible candidate rather than an isolated analytic finding.

The signaling readouts were concordant with the lipidomic data. Post-sevoflurane EV exposure increased p-EGFR, PKCα, and p-NF-κB p65 in recipient HeLa cells. An important mechanistic consideration concerns the relationship between DG accumulation and EGFR activation. In canonical EGFR signaling, receptor activation can stimulate phospholipase Cγ activity, leading to DG generation as a downstream second messenger [29]. Because PLC activity, DG manipulation, receptor binding, and pathway inhibition/rescue experiments were not performed, the present data do not establish that DG directly activates EGFR or that DG accumulation is necessary for EGFR phosphorylation. Accordingly, DG enrichment and increased p-EGFR, PKCα, and p-NF-κB p65 should be interpreted as coordinated features of the post-sevoflurane EV-enriched preparation response rather than as evidence of a strictly linear DG-to-EGFR pathway. EGFR is frequently overexpressed in cervical cancer and is associated with aggressive disease biology and poorer prognosis [30,31]. PKCα is a canonical downstream effector of DG-rich membrane signaling [28], and NF-κB is a central regulator of pro-survival and pro-invasive transcriptional programs.

Several limitations should be acknowledged. First, this was a single-center observational study with a modest sample size, and the exploratory follow-up was not powered to detect differences in recurrence or survival. Second, although the phenotypic assays, miRNA profiling, lipidomics, and immunoblot data were internally consistent, causal validation was not performed. Therefore, the observed DG enrichment and alterations in EGFR/PKCα/NF-κB signaling should be interpreted as coordinated mechanistic features rather than a proven signaling pathway. Third, lipidomic and signaling analyses were intentionally restricted to the phenotype-positive sevoflurane arm because post-propofol EVs did not induce measurable changes in the functional assays. That design was reasonable for discovery, but it sets a clear boundary: the current data identify a positive mechanistic signal associated with sevoflurane-derived EVs, whereas subtler metabolic effects of propofol-derived EVs cannot be excluded. Fourth, functional validation was conducted in HeLa cells and HUVECs. Although these models provided a controlled and interpretable experimental system, the cervical cancer-specific findings were derived from a single cervical cancer cell line. Given the biological heterogeneity of cervical cancer, the extent to which these observations apply to other cervical cancer subtypes remains uncertain. Validation in additional cervical cancer cell lines and primary patient-derived cervical cancer cells will be important to establish the broader generalizability of the present findings. Fifth, the plasma EV-enriched preparations were obtained by differential ultracentrifugation without additional purification procedures and without assessment of lipoprotein-associated markers. Therefore, co-isolation of circulating lipoproteins or other non-EV particles cannot be formally excluded. In addition, lipidomic analyses were performed in recipient HeLa cells after exposure to EV-enriched plasma fractions rather than by direct profiling of the isolated preparations. The observed lipid alterations should therefore be interpreted as recipient-cell remodeling responses and not as definitive measurements of EV lipid cargo. These considerations should guide interpretation of the present findings.

Taken together, the present study does not support direct clinical claims regarding anesthetic selection and survival, but it does identify a biologically coherent perioperative EV signal in cervical cancer. Plasma EVs collected after sevoflurane anesthesia were associated with enhanced malignant phenotypes in recipient cells, DG-centered lipid remodeling, and increased EGFR/PKCα/NF-κB signaling. These observations define a focused mechanistic framework for future validation studies and support further investigation of perioperative EV biology as a potential determinant of tumor behavior in cervical cancer.

## 5. Conclusions

Plasma EVs collected after sevoflurane anesthesia were associated with enhanced aggressive phenotypes in recipient cervical cancer cells and with lipid remodeling characterized by DG enrichment and increased EGFR/PKCα/NF-κB signaling. These findings support an exploratory mechanistic framework linking perioperative EV biology to cervical cancer cell behavior and warrant further causal validation in broader experimental models.

## Data Availability

The miRNA sequencing data have been deposited in the NCBI Sequence Read Archive (SRA) and are accessible under BioProject accession number PRJNA1456551. The lipidomics data have been deposited in the OMIX database, China National Center for Bioinformation/Beijing Institute of Genomics, Chinese Academy of Sciences (https://ngdc.cncb.ac.cn/omix (accessed on 8 June 2026)), under accession number OMIX016465.

## Supplementary Materials

The following supporting information can be downloaded at: https://www.mdpi.com/article/10.3390/biomedicines14061333/s1, Table S1: Comparison of baseline clinical features between the propofol and sevoflurane group; Table S2. Primers used for qRT-PCR; Figure S1: Survival outcomes of cervical cancer patients receiving propofol or sevoflurane anesthesia.

## Abbreviations

The following abbreviations are used in this manuscript:

EV	Extracellular vesicle
PBS	Phosphate-buffered saline
HUVECs	Human umbilical vein endothelial cells
FDR	False discovery rate
KEGG	Kyoto Encyclopedia of Genes and Genomes
RT-qPCR	Reverse transcription quantitative PCR
OPLS-DA	Orthogonal partial least squares-discriminant analysis
TEM	Transmission electron microscopy
