# Supplementary material for: Sevoflurane-Associated Plasma Extracellular Vesicles Promote Aggressive Phenotypes in Cervical Cancer Cells with Concurrent DG Remodeling and EGFR/PKCα/NF-κB Activation
Source: Biomedicines. 2026 Jun 12;14(6):1333. doi: 10.3390/biomedicines14061333 (PMC13297343; doi:10.3390/biomedicines14061333)
Supplement: Supplementary file 1 [file biomedicines-14-01333-s001.zip › biomedicines-4305890-supplementary.pdf]

Table S1. Comparison of baseline clinical features between the propofol and sevoflurane group.

	Participants (n=53)		
	sevoflurane (n=28)	Propofol (n=25)	*P* value
Age, year, median (p25-p75)	53(44-57)	52(46-57)	0.972
BMI, kg/m^2, mean±SD	23.3±2.2	23.2±2.1	0.864
Tumor stage, n(%)			0.748
Stage IIa	18(64.3)	15(60.0)	
Stage IIb	10(35.7)	10(40.0)	
Squamous cell carcinoma antigen, μg/L, median (p25-p75)	1.1(0.95-2.9)	1.0(0.8-1.3)	0.166
Duration of anesthesia, min, median (p25-p75)	247(242-267)	245(232-260)	0.367
Sufentanil, μg, median (p25-p75)	30(30-40)	30(30-35)	0.802
Remifentanil, μg, median (p25-p75)	2.0(2.0-2.5)	2.0(2.0-2.0)	0.667
Fluid administration, L, median (p25-p75)	1.0(1.0-2.4)	1.5(1.0-2.0)	1.000
Minimum body temperature, °C, mean±SD	36.0±0.2	36.0±0.1	0.690

Table S2. Primers used for qRT-PCR.

**Primer**	**Sequence (5′-3′)**	**Species**
hsa-miR-130a-3p (RT)	GTCGTATCCAGTGCAGGGTCCGAGGT ATTCGCACTGGATACGACATGCCC	*Homo sapiens*
hsa-miR-130a-3p-F	CGCGCAGTGCAATGTTAAAA	
hsa-miR-130a-3p-R	AGTGCAGGGTCCGAGGTATT	
hsa-miR-93-5p (RT)	GTCGTATCCAGTGCAGGGTCCGAGGT ATTCGCACTGGATACGACCTACCT	*Homo sapiens*

hsa-miR-93-5p-F	CGCAAAGTGCTGTTCGTGC	
hsa-miR-93-5p-R	AGTGCAGGGTCCGAGGTATT	
hsa-miR-328-3p (RT)	GTCGTATCCAGTGCAGGGTCCGAGGT ATTCGCACTGGATACGACACGGAA	*Homo sapiens*
hsa-miR-328-3p-F	GCTGGCCCTCTCTGCCC	
hsa-miR-328-3p-R	AGTGCAGGGTCCGAGGTATT	
hsa-let-7d-5p (RT)	GTCGTATCCAGTGCAGGGTCCGAGGT ATTCGCACTGGATACGACAACTAT	*Homo sapiens*
hsa-let-7d-5p-F	GCGCGAGAGGTAGTAGGTTGC	
hsa-let-7d-5p-R	AGTGCAGGGTCCGAGGTATT	
hsa-let-7g-5p (RT)	GTCGTATCCAGTGCAGGGTCCGAGGT ATTCGCACTGGATACGACAACTGT	*Homo sapiens*
hsa-let-7g-5p-F	CGCGCGTGAGGTAGTAGTTTGT	
hsa-let-7g-5p-R	AGTGCAGGGTCCGAGGTATT	
hsa-let-7i-5p (RT)	GTCGTATCCAGTGCAGGGTCCGAGGT ATTCGCACTGGATACGACAACAGC	*Homo sapiens*
hsa-let-7i-5p-F	CGCGCGTGAGGTAGTAGTTTGT	
hsa-let-7i-5p-R	AGTGCAGGGTCCGAGGTATT	
U6 (RT)	GTCGTATCCAGTGCAGGGTCCGAGGT ATTCGCACTGGATACGACCACGCAA	*Homo sapiens*
U6-F	CTCGCTTCGGCAGCACA	
U6-R	AACGCTTCACGAATTTGCGT	

Supplementary Figure

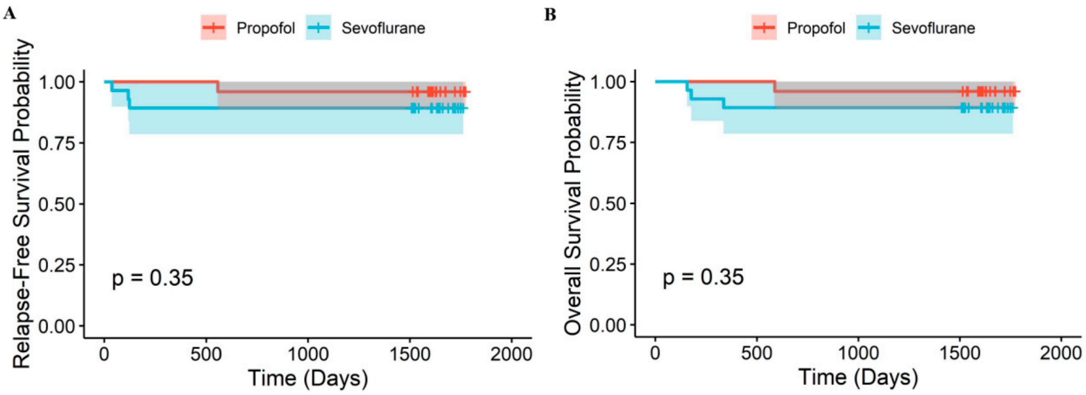

Figure S1. Survival outcomes of cervical cancer patients receiving propofol or

sevoflurane anesthesia. (A) Disease-free survival (DFS) and **(B)** overall survival (OS) rates over 1500 days post-surgery. No significant differences were observed between the propofol (n=25) and sevoflurane (n=28) groups (log-rank test, $P > 0.05$).
